# Atopic Dermatitis – Current State of Research on Biological Treatment

**DOI:** 10.34763/jmotherandchild.2020241.2003.0000010

**Published:** 2020-07-29

**Authors:** Barbara Klasa, Ewa Cichocka-Jarosz

**Affiliations:** 1 Department of Pediatrics, Jagiellonian University Medical College, Kraków, Poland

**Keywords:** atopic dermatitis, biological treatment, dupilumab, interleukin inhibitors, JAK-STAT inhibitors, phosphodiesterase-4 inhibitors

## Abstract

Atopic dermatitis (AD) is the most common atopic disease in young children and most common skin disease in childhood. In the Polish population, the incidence of AD in the group of children aged 6–14 is about 4% and it is underestimated. The disease is chronic and recurrent, and the leading symptom is skin pruritus that in the mechanism of the vicious circle is accompanied by scratching that causes generalized infections. The overall problems lead to a decrease in the quality of life of the child and its parents and to an increased risk of psychosomatic diseases. The complex pathomechanism of AD is due to chronic inflammation of the skin, in which various cell phenotypes are involved. The management is comprehensive and it is aimed at reducing inflammation, improving the skin barrier function, reducing the symptoms of dryness and itching of the skin and secondarily improving the quality of life. The treatment includes intensive skincare, anti-inflammatory treatment based on the proactive use of topical glucocorticosteroids and topical calcineurin inhibitors. Periods of exacerbation of lesions require intensified treatment. In particularly severe, recurrent cases, treatment options can be extended to systemic immunosuppressive drugs, with awareness of their adverse effects. Previous year has brought significant progress in the current treatment of AD in the form of biological treatment. Cytokines and other mediators that play an important role in the pathogenesis of skin inflammation have become a target for new forms of therapy. Drugs for which interleukin (IL)-4 and IL-13 are the targets are particularly represented. Dupilumab is the first biological drug approved for the general treatment of children aged >12 years with moderate to severe AD. Another therapeutic option for topical use is crisaborole, a phosphodiesterase-4 inhibitor. This study presents the current state of research on biological drugs in AD.

## Introduction

Atopic dermatitis (AD) is the most common atopic disease in small children and a leading skin disease during childhood. AD affects 7–30% children and 1–10% adults depending on the type of population or region of the world. Disease characteristics are heterogeneous and they vary by region and age (1). Large cohort studies indicated that AD occurs in 11–16% patients in the European population (2). In the Polish population, according to the results of ECAP (*Epidemiologia Chorób Alergicznych w Polsce* [Epidemiology of Allergic Diseases in Poland]) in the questionnaire survey, where in some children the result of the survey was verified by the medical examination, the frequency was 3.91, 4.3 and 3.02%, respectively, in the examined age groups (6–7 years, 13–14 years and adults), though this frequency seems to be underestimated (3).

The disease is chronic and recurrent with the primary symptoms resulting from the skin barrier dysfunction accompanied by intense itching and characteristics of the chronic skin inflammation. The environmental (emotions, sweating and physical exercise) and microbiological factors (skin colonization with *Staphylococcus aureus* common in these patients) play an important role in the disease flare-ups, leading to disturbances in sleep and concentration. The course of the disease warrants necessity of constant employment of time-consuming procedures and treatment of recurrent flare-ups. These result in, regardless of patient's age, reduced quality of life, increased risk of psychosomatic illnesses such as attention-deficit hyperactivity disorder and depression (4). In case of children, the above chronic illness leads to reduced quality of life and disturbances in life of the entire family (5).

**Figure 1:**
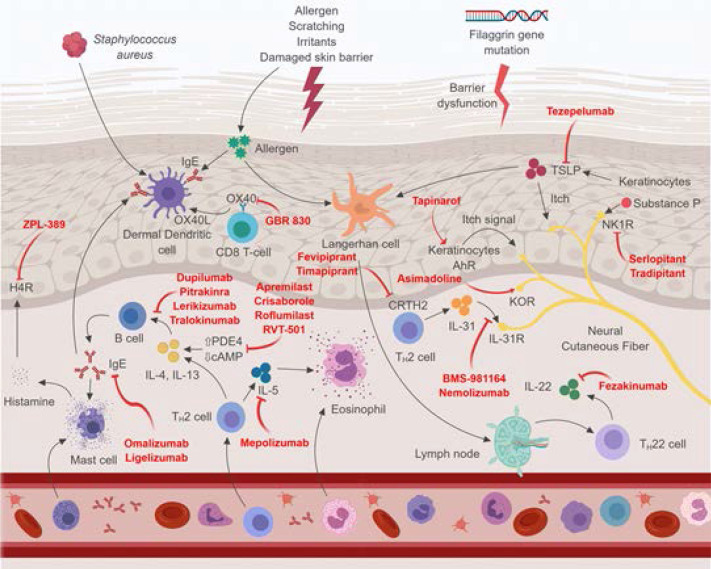
The pathomechanism of inflammation and current research directions for biological treatment of AD (courtesy of HL Nguyen, MM Tollefson, with permission from the Springer Nature publishing house) [10]

The pathogenesis of AD is complex, and it results from the chronic inflammatory status in skin which is associated with different phenotypes of lymphocytes (i.e. Th2, Th17, Th22 and Th1), other competent immunological cells (innate lymphoid cells [ILC] and dendritic cells [DC]) or cells of the skin barrier (keratinocytes). The released cytokines, such as IL-4, IL-13, IL-17, IL-22, IL-31 and thymic stromal lymphopoietin (TSLP), form a network of mutual interactions, sustain chronic inflammatory state, weaken barrier function of the skin and play a role in disease flares (Table 1) (6, 7). The key role of cytokines is associated with Th2 (IL-4, IL-5 and IL-13) (8, 9). Taking into account biological markers and current research directions on biological treatment in AD targeted at these markers, the pathomechanism of inflammation is shown in Figure 1 (10).

**Table 1:** Characteristic changes in acute and chronic atopic dermatitis (AD)

**Characteristic**	**Exacerbation AD**	**Chronic form AD**
Th2 type activation pathway and related cytokines/chemokines	Increased activity of IL-4, IL-13, IL-31	Increased activity of IL-5, IL-13, IL-31, IL-10, CCL5, CCL13, CCL18. Equivocal results for IL-4
Th22 type activation pathway and related cytokines	Increased IL-22 activity	Increased activity of IL-22, IL-32
Th1 type activation pathway and related cytokines/chemokines	A slight increase in IFN-g, MX1, IL-1b, CXCL9-11, but not in all phenotypes	Significant increase in IFNg, MX1 (markers associated with Th-1 cytokine), IL-1b, CXCL9-11
Th17 type activation pathway and related cytokines	A slight increase in the level of IL-17, IL-23p19, IL-23p40	The level of activation is similar to that of acute AD
Infiltration of immune cells	Infiltration of immune cells	Intensification of changes as in the exacerbation
Epidermal changes	Increased hyperplasia, epidermal thickening, marker proliferation (Ki67, K16, IL-22); reduction in the level of epidermal barrier proteins (involucrine, loricrin, filaggrin)	Intensification of changes as in the exacerbation
Reduced expression of final protein and lipid differentiation	Decreased expression of FLG, LOR, PPL and other differentiation proteins; significant lipid disorders	Intensification of changes as in the exacerbation

*Sources*: Moyle et al. (6) and Suárez-Fariñas et al. (7).

AD in childhood is very heterogeneous. There is a continuing search for predictors defining a phenotype of this disease. In one of the large cohort studies, the strongest predictors of association with a particular phenotype (cluster) were variables such as age of the first symptoms, age at the time of diagnosis, number of white blood cells and eosinophils and total IgE concentration (11). These parameters allowed to identify four phenotypes: A (early beginning of the symptoms <2 years old, eosinophilia, increased total IgE concentration, allergy to food allergens and high points in scoring atopic dermatitis [SCORAD] scale), B (early beginning of the symptoms <2 years old, low eosinophilia, low total IgE concentration and low IgE specific for food and airborne allergens), C (early beginning of the symptoms and increased values of CRP and white blood cells) and D (beginning of the symptoms >2 years old, increased total IgE concentration and high concentration of IgE specific for airborne allergens) (11). Regardless the attempts to identify phenotypes, the natural course of the disease is characterized by a chronic inflammatory state with overbearing skin dryness and lichenified lesions, periods of flare-ups accompanied by inflammation-related parameters such as intense redness, oedema, increased inefficiency of the skin barrier, oozing, intense pruritus and pain. The chronic phase is associated with predominance of lymphocytes Th1, Th17 and their cytokines (interferon and IL-17A) (12).

In case of flare-up, there is an increased expression of lymphocytes Th2, Th22 and their cytokines (IL-4, IL-13 and IL-22), as well as DCs (12). IL-4 and IL-13 are the key cytokines leading to local increase of IgE levels, a part of which is characterized by autoreactivity against their own antigens. Both cytokines play significant role in pruritus. In both types of inflammation states, there is a type of ‘vicious circle’ where IL-4, IL-13 and IL-22 inhibit the expression of filaggrin (FLG), which worsens problems of the skin barrier. Keratinocytes release inflammatory mediators, such as TSLP, which in turn stimulates Th2 response and promotes production of IL-13 and IL-31, and those in conjunction with nerve growth factor (NGF), and substance P represents strong pruritus mediators. Scratching, secondary to intense itchiness, worsens dysfunction of the epidermal barrier, while anxiety and psychosomatic discomfort increase experience of itchiness that stimulates more scratching (Table 1) (6, 7).

## Treatment

Management of AD is complex, and its main goal is to decrease inflammatory status, improve skin barrier function and decrease skin dryness and pruritus resulting in improvement of quality of life. Treatment includes intensive skincare, anti-inflammatory treatment based on topical corticosteroids (TCS) with weak to moderate strength of action and topical calcineurin inhibitors (TCI). Periods of flare-ups require intensified treatment, which is mostly based on the TCS (13). In some cases, the reason behind unsuccessful treatment might be fear of caregivers and/or physicians of steroid therapy (14). Systemic corticosteroids (CS) are not recommended in AD treatment; however, they are still used in the clinical practice despite risks outweighing benefits of such treatment (15).

Treatment in some particularly serious, recurrent types of disease might also include immunosuppressive systemic medications, such as cyclosporine A (CsA), methotrexate (MTX) or azathioprine, keeping in mind their associated adverse effects. This group of medications is hardly ever administered in children. The medications with the well-documented efficacy and safety include CsA (16) and less common MTX (17). The indispensable element of treatment includes education of parents/caregivers in prophylaxis of exposure to factors worsening skin status, appropriate types skincare and external type of management (Table 2) (18, 19). Aside from introduction of TCI, there was no breakthrough in treatment of AD since many years.

**Table 2:** Strategy for gradual treatment of atopic dermatitis (AD)

	**Chronic treatment**	**Treatment of exacerbations (often recommended in a hospital setting)**
Severity III SCORAD^3^ 50	1st line: dupilumab, cyclosporin A, phototherapy (in selected cases) 2nd line: methotrexate, azathioprine Periodic intensification of basic treatment such as emollient for the skin under a wet dressing	Intensification of basic treatment such as emollient for the skin under a moist dressing Systemic glucocorticosteroid (GCS) (short – maximum up to 14 days) and/or local GCS daily all over the skin (possibly for several under the dressing) **Limitation of pyodermisation** Antibiotic in general (first generation cephalosporins or clindamycin preferred) Antibiotic topically (fusidic acid, mupirocin, retapamulin) Mupirocin locally on the skin of the atrium of the nose in attempts to eradicate the carrier of *S. aureus* Disinfectants (polidocanol, octenidine, chlorhexidine, triclosan, KMnO_4_) and desiccants (tannin preparations) **Herpes infection** Acyclovir and observation in a hospital setting **Yeast or dermatophyte infection** Consider systemic treatment with imidazole derivatives (fluconazole >1 year old, itraconazole >12 years old), miconazole, terbinafine **Molluscum contagiosum infection – usually self-limiting** Technological fabrics with silver ions in some patients
Severity II SCORAD^3^ 25–49	Local GCS with weak to moderate proactive strength Proactive calcineurin (Protopic, Elidel) or PhD4 (Eucrisa) topical inhibitors Periodic intensification of basic treatment such as emollient for the skin under a wet dressing	
Severity I SCORAD < 25	Local GCS with weak proactive strength Topical calcineurin inhibitors (Elidel) proactively or PhD4 (Eucrisa)	
Basic treatment for all levels of severity AD	Avoiding irritants (e.g. rough fabrics) and aggravating (sweating, overheating, stress) Dermocosmetics for washing the skin Emollients ^3^ 2 times per day used generously (after washing according to the principle of up to 5 min) Elimination diet in justified cases Probiotics with documented effects Symptomatic antipruritic drugs	

*Sources*: Klasa and Cichocka-Jarosz (18), own modification based on Brunner et al. (19).

## New age in therapy, biological treatment, personalized therapy

The medications targeted against mediators of inflammation, such as cytokines (mostly Th2 profiles such as IL-4, IL-13, TSLP, IL-25, IL-31 and IL-33), enzymes (Janus kinase and phospholipase A2), receptors (receptors specific for aryl carbohydrates) and skin microbiota, are the targets for the potential new therapies in the treatment of AD. The recent years brought considerable progress in the existing treatment options, opening up possibilities to treat serious, recurrent types of disease based on the biological agents (10). Even though the majority of those medications is addressed to the patients regardless of their AD phenotype, it is possible that as in asthma, biological treatment might become personalized, targeted against specific markers and adjusted to particular phenotypes (pattern of symptoms), endotypes (pattern of markers specific for particular pathology) or teratypes (pattern of response to specific treatment) of the disease.

Currently, as part of personalized treatment, there are attempts to propose new treatments that target cytokines and other mediators are playing significant role in the pathogenesis of the skin inflammatory status. There is a special group of medications that target IL-13. However, some of them, despite their potentially beneficial effect in regard to their pathophysiology/type of mechanism, did not show expected results in the treatment of AD in children, i.e. medications blocking interleukins (ILs), such as IL-5 and IL-22, and prostaglandin D_2_ receptor 2 (CRTh2) (6, 7, 12, 19).

## IL-4/IL-13 inhibitors

Potential efficacy of monoclonal antibodies specific for IL-13 results from its principal role in development of the inflammation. In skin biopsy in children with AD, there was increased expression of IL-13 mRNA compared with the skin of healthy children, and the level of IL-13 mRNA expression was proportional to the severity of the disease. Moreover, overexpression of IL-13 is the cause of the decreased epidermal integrity by deregulation of the key components of the skin components. Treatment with systemic medications such as CsA might lead to decrease in IL-13 level, and the current results of the studies on dupilumab, an antibody specific for IL-13/IL-14, confirm key role of IL-13 in the development of inflammation. Therefore, the targeted treatment aimed against the most central point in AD pathology might maximize effectiveness of the treatment and may limit its toxicity (20).

### Dupilumab

Treatment options in patients diagnosed with moderate or severe AD used to be limited. The first biological medication, approved for treatment of adults and children over 12 years, diagnosed with moderate and serious type of AD is dupilumab (Dupixent^®^, Sanofi-Aventis Groupe, Paris). It is a monoclonal antibody blocking subunit alpha, a common one for the receptors of IL-14 (IL-4Ra) and IL-13 (21). Cytokines such as IL-4 and IL-13 exert a direct key influence on the epidermis of the patients with AD by (1) blocking terminal differentiation with potential hyperplasia of the regulatory loop (hyperprolifieration of keratinocytes), (2) stimulation of remodelling of the skin towards sponge layer, (3) inhibition of lipid synthesis in the skin, (4) reduced synthesis of antimicrobial peptides (22) and (5) facilitation of the skin colonization by *S. aureus* (23).

The results in the key phase 3 of the (clinical) study to determine the effectiveness of dupilumab in monotherapy in patients over 12 years with uncontrolled AD (ClinicalTrials.gov: NCT03345914) were comparable with the adult population (ClinicalTrials.gov: NCT02277769, NCT02277743):

- In the Eczema Area and Severity Index (EASI) scale, there was a triple improvement in comparison with the placebo group, 66% vs 24%, respectively.
- There were above 10 times more treated patients who achieved a complete remission of the skin changes compared with the placebo group, 24% vs 2%, respectively.
- In the EASI-75, there were five times more patients who noticed improvement of their skin state by at least 75% compared with the placebo group, 42% vs 5%, respectively.
- There were above seven times more patients who reported decreased pruritus compared with the placebo group, 37% vs 5%, respectively.
- There was a significant decrease in the serum inflammatory biomarkers, including those specific for IgE allergens in the patients treated with dupilumab.

According to EMA Pediatric Committee decision (Opinion of the Paediatric Committee on the acceptance on the modification of an agreed Paediatric Investigation Plan, EMA/PDCO/92908/2018), there is an agreement for conducting the clinical trials dedicated to the evaluation of safety and efficacy of dupilumab in patients aged over 6 months till 18 years (24). Currently in children aged between 6 and 12 years, and 6 months and 6 years, with AD, phase II–IV clinical studies were initiated (ClinicalTrials.gov: NCT02407756, NCT02612454, NCT03411837, NCT03428646, NCT03549416). Finally in pre-registration data, dupilumab was evaluated in >30 clinical studies in >7,000 patients aged over 12 years. Safety profile of dupilumab used in children over 12 years for 52 weeks was appraised similarly to the studies in adult populations (25). So far, all the results of the studies on dupilumab demonstrated high safety profile, good drug tolerability and lack of dose-limiting toxicity. The adverse effects encountered during treatment, such as conjunctivitis, eyelid inflammation, oral herpes, eosinophilia, rhinitis, pharyngitis, headaches and upper respiratory tract infections, were very few and almost negligible (21). Dupilumab is manufactured in two doses at 200 and 300 mg for subcutaneous route. Initially drug was registered by Food and Drug Administration (FDA) in the USA for adults (29 March 2017) and subsequently for adolescents (11 March 2019), followed by European Medicines Agency (EMA) approval in the European Union (EU) (6 August 2019). As of 26 September 2017, Dupixent at the dose 300 mg (pre-filled syringe 150 mg/ml and auto-injector 150 mg/ml) is registered in Poland for use in the treatment of moderate to severe AD in adults and currently also in adolescents aged 12 years and older who are eligible for general treatment.

Up-dosing schedule includes as follows: in adults, initiating dose equals 600 mg (separated into two injections), followed by 300 mg every 2 weeks; in teenagers over 12 years, the dose depends on body weight – in those of body weight <60 kg the initial dose equals 400 mg (two injections of 200 mg), followed by 200 mg fortnightly, and in those of body weight >60 kg the schedule is the same as in adult patients (24, 25).

### Tralokinumab

Tralokinumab is a monoclonal antibody that strongly blocks association of IL-13 with the receptors specific for IL-13Ra1 and IL-13Ra2 and disrupts signal transduction to the receptors IL-4Ra/IL-13Ra1. The goal of the clinical studies was the analysis of efficiency and safety of tralokinumab in monotherapy (ClinicalTrials.gov: NCT03131648, NCT03160885) or in combination with TCS (ClinicalTrials. gov NCT03363854, NCT03761537) in adults with moderate to serious AD. In phase II study there were 204 participants, randomized into four arms 1:1:1:1 who received tralokinumab at the doses 45, 150 and 300 mg or placebo, respectively, every 2 weeks for 12 months. Along with that treatment, concurrent application of the TCS was served. The final results showed that 300 mg of tralokinumab resulted in a significant reduction of AD symptoms compared with the placebo group, evaluated by means of both EASI and SCORAD scores (ClinicalTrials.gov: NCT02347176) (26). At the next stage, the clinical study evaluating monotherapy with tralokinumab for adolescent subjects with moderate to severe AD was initiated (ClinicalTrials.gov: NCT03526861, NCT03562377).

### Lebrikizumab

Lebrikizumab is a humanized monoclonal antibody with a high affinity for IL-13. The results of the studies so far verified that lebrikizumab at a dose of 125 mg applied subcutaneously every 4 weeks, combined with application of TCS, led to reduced need for immunosuppressive drugs in the patients with AD. In phase II study, lebrikizumab used as an additional therapy to the TCS in adults showed better results compared with the patients receiving placebo and TCS (ClinicalTrials.gov: NCT02465606, NCT04178967, NCT04146363) (20). The next step is the ongoing study assessing the safety and efficacy of lebrikizumab in adolescent patients with moderate to severe AD (ADore) (ClinicalTrials.gov: NCT04250350, NCT04250337).

### Pitrakinra

Pitrakinra is a human recombinant variant (mutein) of IL-4 which binds subunit alpha of IL-4 and blocks activity of IL-4 and IL-3; therefore, it prevents development of inflammation induced by those cytokines. Animal models of asthma and AD show that pitrakinra exerts strong blocking effect on proliferation mediated by IL-4 and IL-13, and it reduces allergens-induced inflammation. In phase I and II studies in patients with asthma, pitrakinra administered by either subcutaneous injection or by inhalation decreased inflammation in the respiratory tract.

In 2007, the drug was approved to treat patients with asthma (27). Phase IIa of the clinical study to verify efficacy of pitrakinra (Aeroderm) compared with placebo in the patients with moderate and serious type of AD was completed (ClinicalTrials.gov: NCT00676884). Pitrakinra administered subcutaneously showed some promising results in the treatment of AD; however, the results are not yet published. Further research is required to accurately determine the value, characteristics, effectiveness and its safety.

## Inhibitors of IL-31/IL-31Ra

IL-31 is a cytokine released by macrophages, dendritic cells, eosinophils, basophils, mastocytes, keratinocytes and Th2 cells. Association of IL-31 with its receptor alpha (IL-31Ra) on the immunological cells, keratinocytes and neural fibres results in the activation of Janus kinase and Signal Transducer and Activator of Transcription (JAK/STAT), which plays important role in the development of pruritus (28). Prevention of IL-31 binding to IL-31Ra might significantly reduce itching in patients with AD.

### Nemolizumab

Nemolizumab (CIM3312) is a monoclonal antibody specific for IL-31 receptor, hence it blocks signal transmission mediated by IL-31. IL-31 is a key cytokine that regulates pruritus, which worsens epithelial dysfunction leading to easier access of allergens (29). Until present, there are two clinical studies to determine efficacy and safety of nemolizumab. Phase I study showed significant decrease in pruritus according to EASI scale. In phase II study, safety and tolerability of the medicine were tested. The studies showed that nemolizumab was well tolerated, and it decreased intensity of the pruritus, skin inflammation and sleep disturbances compared with placebo control. However, the results in EASI, SCORAD and body surface area (BSA) scales were not significantly different between the study and control groups after 12 weeks of treatment. Increasing duration of the treatment with nemolizumab up to 64 weeks resulted in decreased skin itching; however, the results were not compared with the control group and therefore the results could not be verified. The most common acute adverse effects of the treatment observed in >2% of the patients randomized to receive nemolizumab included worsening of AD (8%), upper respiratory infection (4%), rhinitis with pharyngitis (4%), peripheral oedema (3%), increased activity of creatine kinase (3%) and local reactions at the site of injection (2%). Currently, there are phase I–III studies in the patients older than 6 years (ClinicalTrials.gov: JapicCTI-173740, JapicCTI-183894, NCT03100344).

The primary outcome of the nemolizumab study was reduction in skin itching intensity following 12 weeks of treatment as determined by the visual analogue scale (VAS). The secondary outcome was the level of skin itching intensity as determined by VAS and SCORAD scale (30). There were 264 patients enrolled in part A, out of them 216 completed the study. There were 191 patients enrolled in part B, out of them 131 completed the study. The patients enrolled in part A suffered from more intense pruritus according to VAS, and moderate to serious AD according to IGA, BSA and EASI scales. The most common allergic illness in those patients was allergic rhinitis (*n* = 91), followed by asthma (*n* = 34) patients. The most spectacular improvement was observed in the group, which received nemolizumab at 0.5 mg/kg. The first results were observed during 16th week of treatment that corresponded to 4th week of treatment with the active ingredient in the patients who received placebo for 12 weeks during part A followed by nemolizumab. There were no safety-related adverse effects associated with long-term nemolizumab use (30).

## Inhibitors of IL-12 and IL-23

### Ustekinumab

Ustekinumab is a human monoclonal antibody specific for IL-12 and IL-23. IL-23 is indispensable for the generation of Th-17 cytokine, which plays a key role in the pathogenesis of inflammation. In a mouse model, it was demonstrated that IL-23 mediated inflammation typical for AD, and IL-23 level was proportional to degree of severity of the AD symptoms. The results of ustekinumab usefulness in the treatment of AD are barely convincing (ClinicalTrials.gov: NCT01806662, NCT01945086). In some cases, ustekinumab seemed to be beneficial; however, the treatment results were either very weak or absent in the remaining cases (31).

## Inhibitors of JAK-STAT

JAK-STAT is a pathway for signal transduction from the cellular membrane to the nucleus. It regulates immunological system by pro-inflammatory cytokines, such as IL-4, IL-5, IL-13, IL-31 and TSLP. Association of the ligands with their receptors in the cell membrane leads to JAK-STAT activation; therefore, inhibition of this complex blocks the development of inflammation (10). JAK inhibitors administered orally or locally appear to provide a promising therapeutic option for adults and children with AD, demonstrating high efficacy in comparison with placebo controls. In addition, they result in significant reduction in skin itching. Those drugs are well tolerated with very few side effects (10).

### Baricitinib

Baricitinib is an oral JAK1/JAK2 inhibitor which was tested only in the adult population so far. There were 124 patients in phase II clinical study. There were significantly more patients who achieved improvement by >50% compared with the initial skin condition according to EASI-50 scale in the treated group in comparison with placebo, 61% vs 37%, *p* = 0.027 (32). All the patients included in this study used TCS for a month preceding baricitinib, hence its efficacy in monotherapy was unknown. The recorded side effects included lymphopenia, neutropenia, worsening of AD, headaches, without any other serious symptoms. Currently, there are phase III studies in adults to determine safety and efficacy of baricitinib, along with its efficacy in monotherapy (ClinicalTrials. gov: NCT03334396, NCT03334435, NCT03435081, NCT03559270, NCT03334422, NCT03428100).

### Upadacitinib

Upadacitinib is an oral JAK1 inhibitor tested in children aged 2–17 years and in adults with AD. Phase IIB studies in adults showed better efficacy of upadacitinib compared with placebo (*p* < 0.001) (31). The most common side effects were upper respiratory tract infections (33). Currently, efficacy of upadacitinib is analysed in phase III study in youths and adults (ClinicalTrials.gov: NCT03569293, NCT03568318, NCT03607422, NCT03661138), while phase I study was just launched in children aged 2–12 years (ClinicalTrials.gov: NCT03646604). Guttman-Yassky et al. presented results of phase II study in adults with AD which evaluated upadacitinib efficacy by serum IgE levels and eosinophilia (33). During the study, the patients received upadacitinib 7.5, 15 and 30 mg or placebo daily. According to EASI scale, the average percentage of improvement from the initial condition to 16th week of treatment was significantly higher in all the patients treated with upadacitinib regardless of their dose compared with placebo.

### Abrocitinib

Abrocitinib (Pf-04965842) is an oral JAK1 inhibitor which is characterized by very few side effects, out of which the most common ones include upper respiratory tract infections and AD flare-ups. Phase II studies in adults verified efficacy and good tolerance of the drug in the patients with moderate and serious AD (34). Currently, phase III study is to determine the efficacy of the Pf-04965842 in youth over 12 years (ClinicalTrials.gov: NCT03575871, NCT03349060, NCT03422822, NCT03627767).

### Ruxolitinib

Ruxolitinib is a locally acting JAK1/JAK2 inhibitor, initially used in myelofibrosis and polycythemia vera. Currently, it is tested in adults and children over 12 years with AD. Phase IIB studies in adults initially fulfilled all the primary and secondary aims (35). Currently, phase I study is carried out in children aged 12–17 years (ClinicalTrials.gov: NCT03257644).

### ASN002

ASN002 is an oral inhibitor of JAK, tyrosine kinase 2 (TYK2) and spleen tyrosine kinase (SYK). Inhibition of SYK disrupts the function of B cells, mastocytes, macrophages, IL-17, Th1, Th2 and Th17, and simultaneously it regulates differentiation of keratinocytes. All the adult patients included in this study reached EASI-50 after 4 weeks of treatment (ClinicalTrials. gov: NCT03139981) (36).

### Delgocitinib

Deglocitinib is a local inhibitor of JAK/TYK2 currently tested in children aged 12–17 years, adults with AD and adults with hand eczema (ClinicalTrials.gov: NCT0372572, NCT03826901, NCT03683719). The observed side effects included pharyngitis, lymphopaenia and erysipelas (37).

## Mild and moderate AD

### Topical inhibitors of phosphodiesterase-4

In patients with AD, there is an elevated level of phosphodiesterase-4 (PDE-4) in the lymphocytes. PDE-4 is an intracellular enzyme associated with synthesis of pro-inflammatory cytokines, which plays a role in inflammatory pathways. Its inhibition blocks inflammatory cytokines and inflammation development in the skin. PDE-4 inhibition became a chance for new type of local treatment (38).

One of the issues regarding current local treatment of AD is associated with safety of its long-term use. Until recently, the approved methods of local treatment included TCS and TCI. However, despite acceptable treatment results, the recommendations do not advice prolonged use of high concentrations of TCS because they might lead to serious adverse effects, such as local skin atrophy and teleangiectasia, or systemic outcomes due to dermal drug absorption, such as inhibition of hypothalamus–pituitary axis. Since AD is a chronic and relapsing disease, it requires continuous treatment with the least number of possible adverse effects. There was no new treatment option approved for the local use in the past 15 years.

### Crisaborole

Crisaborole is a non-steroid, anti-inflammatory inhibitor of PDE-4. The drug as 2% ointment blocks PDE-4-dependent degradation of adenosine cyclic monophosphate. The results of the preclinical studies verified effective penetration of crisaborole through the skin. In addition, the drug is quickly metabolized to inactive metabolites, which determines the safety of its use. Phase III study demonstrated very few side effects of crisaborole ointment use (ClinicalTrials.gov: NCT04040192, NCT02118766). It appears to be the next therapeutic option in prolonged local treatment of AD, with the treatment potential comparable with pimecrolimus (38). Currently, data from studies on age group 3 months to 2 years are also available (NCT03356977). First, crisaborole (Eucrisa, Pfizer Labs, NY) was approved by FDA in the USA (14 December 2016) for children aged >2 years with mild to moderate AD, but currently in the USA it is registered for children older than 3 months. On 30 January 2020, crisaborole (Staquis, Pfizer Europe MA EEIG) as 20 mg/g ointment was approved by EMA for use in the EU in adults and children aged >2 years.

### RVT-501

RVT-501 is a local PDE-4 inhibitor which was tested in adults and children aged 2–15 years. Phase I and II studies of 0.2% RVT-501 used for 12 weeks showed improvement according to EASI and SCORAD scores. The side effects included gout, enterocolitis and erythema (39). Phase II study with higher, 0.5% concentration of RVT-501 was completed; however, the results are not published yet (ClinicalTrials.gov: NCT03394677, NCT02950922).

Biological drugs studied in AD and their targets are summarized in Table 3 (11). The list of clinical trials conducted for biological drugs in AD is presented in Table 4 (40).

**Table 3:** Treatment depending on the severity of atopic dermatitis (AD)

**Medication**	**Mechanism of action**	**Characteristics**
AD: mild and moderate form		
PDE-4 inhibitors		
Crisaborole	Inhibits the degradation of PDE4-dependent cyclic adenosine monophosphate, which in turn regulates T-cell signalling pathways, enhancing cellular control of inflammation. Topical drug – 2% ointment	Phase IV studies in children between 3 and 24 months of age have been completed
RVT-501	Phosphodiesterase 4 Inhibitors (PDE4i). Topical drug – 0.5% ointment	Phase II studies in children aged between 2 and 17 years have been completed.
Inhibitors JAK-STAT		
Tofacitinib	Blocking the cell signal transduction pathway inhibits pro-inflammatory cytokines	In the treatment of AD, it has so far only been tested in adults

**AD: moderate and severe form**		

Inhibitors JAK-STAT		
Baricitinib	Inhibitor JAK1/JAK2 – oral drug	All patients included in this study used local GCS 1 month before starting baricitinib therapy, therefore the efficacy of baricitinib monotherapy is unknown
Upadacitinib	Inhibitor JAK1 – oral drug	Currently in the research phase in children aged from 2 to 17 years and adults with AD.
Abrocitinib (Pf-04965842)	Inhibitor JAK1 – oral drug	Currently in phase III studies assessing the effectiveness of the drug in adolescents aged >12 years
Ruxolitinib	Inhibitor JAK1/JAK2 – topical drug	Initially used to treat myelofibrosis and true polycythaemia, it is currently studied in children aged between 12 and 17 years and adults with AD
ASN002	Inhibitor JAK/TYK2/SYK – oral drug	Phase IIa studies in adults aged between 18 and 75 years have been completed.
Delegocitinib	Inhibitor JAK/TYK2 – topical drug	Examined in children aged from 12 to 17 years and adults with AD.

**Phosphodiesterase 4 Inhibitors (PDE4i)**		

Roflumilast	Inhibitor PDE4 – topical drug	Until present, tested only in adults – phase IIa studies showed no improvement after using 0.5% cream Roflumilast
Apremilast	Inhibitor PDE4 – oral drug	Positive results in the treatment of children and adults with refractory AD. High frequency of undesirable activities in the form of cellulitis. No further studies are planned due to the risk and benefit analysis

**CRTH2 receptor antagonists**		

Fevipiprant/Timapiprant	The antagonism on the CRTH2 receptor suppresses the formation of the inflammatory process	Clinical studies have not demonstrated efficacy of the drug relative to placebo (NCT01785602, NCT02002208)
Thymus stromal lymphopoietin (TSLP) and OX40 inhibitors		
GBR-830	Inhibitor TSLP (TSLP induces immune cells to produce pro-inflammatory cytokines). Anti-OX40 monoclonal antibody	Phase II is completed in adults
Tezepelumab	Inhibitor TSLP. Anti-TSLP monoclonal antibody	To date, studies only in adults – phase IIa studies lack satisfactory results
(TAMA) therapeutic aryl hydrocarbon receptor modulating agent (AhR)		
Tapinarof	TAMA	Tested in children aged 12–17 years and adults. Phase III research scheduled for 2019

**Inhibitors IL-4/IL-13**		

Dupilumab	Human monoclonal antibody blocking a subunit, common to IL-4 (IL-4Ra) and IL-13 receptors	To date, dupilumab (Dupixent) has been studied in >7,000 patients aged >12 years, in >30 clinical trials giving very good treatment results (description in the text)
Pitrakinra	IL-4 mutein – binds the IL-4Ra receptor by inhibiting the production of IL-4 and IL-13	Phase II clinical trials in adults have been completed
Tralokinumab/Lebrikizumab	Anti-IL-13 monoclonal antibody	Lebrikizumab and tralokinumab are undergoing phase III studies
Inhibitors IL-5		
Mepolizumab	Anti-IL-5 monoclonal antibody inhibits eosinophil activity	Two clinical trials in adults with AD have been unsuccessful
Inhibitors IL-22		
Fezakinumab	Anti-IL-22 monoclonal antibody	Phase IIa studies have shown that this medicine has little potential to treat AD
Inhibitors IL-12/IL-23 Ustekinumab	Anti-IL-12/IL-23 monoclonal antibody	The results of phase IIa studies are not convincing

**Inhibitors IL-31/IL-31Ra**		

Nemolizumab	IL-31Ra monoclonal antibody	Phase I studies showed a significant reduction of pruritus and phase II studies evaluated the safety and tolerability of the drug. These studies showed that treatment with nemolizumab is well tolerated and it reduces the severity of pruritus, dermatitis and sleep disorders compared with placebo
BMS-981164	Monoclonal antibody IL-31	Phase I studies in 2015 – no results have been published so far
Neurokinin-1 Receptor Antagonists (NK1RA)		
Serlopitant	NK1R antagonist – oral drug	Effective in the treatment of chronic pruritus in adults; however, phase II drug research in patients with AD did not bring satisfactory results
Tradipitant	NK1R antagonist – oral drug	Phase III drug research is currently underway
K-Opioid Receptor Agonists (KOR)		
Asimadoline	Agonist KOR – oral drug	Phase II drug studies have been completed. Effective in reducing itching at night
Histamin Receptor Antagonists-4 (H4R)		
ZPL-389	Antagonist H4R	Studies in adults only. The advantage over placebo has not been demonstrated. Phase IIB study is currently underway
Immunoglobulin E (IgE) inhibitors		
Omalizumab	Anti-IgE monoclonal antibody	There are no satisfactory treatment effects in patients with AD. A drug used to treat asthma
Ligelizumab	Anti-IgE monoclonal antibody	Higher affinity for IgE than omalizumab. Phase II RCT completed

*Source*: Seo et al. (11).

**Table 4:** List of clinical trials conducted for biological drugs in atopic dermatitis (AD)

**Medication**	**Target**	**Study phase**	**Manufacturer**	**www.ClinicalTrials.gov**	**Target endotype**	**AD phenotype**
Dupilumab Dupixent	IL-4Ra	Approved by FDA 2017 – adults, FDA 2019 – adolescents EMA – 2019	Sanofi-Aventis Groupe, Paris	NCT01949311 Phase III in progress – patients >18 years old NCT02407756 Phase II completed – in children 12–18 years old, moderate and severe AD NCT02612454 Phase III in progress – in children ^3^6 months–18 years old NCT03054428 Phase III completed – patients >12–18 years old NCT02407756 Phase II completed Patients ^3^6 to <18 years old NCT02612454 Phase III Patients ^3^6 months old–<18 years old NCT03411837 Phase IV Patients ^3^12 years old NCT03428646 Observation of patients receiving Dupixent Patients ^3^12 years old NCT03549416 New systemic treatment options in patients with AD, including dupilumab (conjunctivitis during dupilumab treatment) Patients: children, adults	Th2/Tc2	All AD phenotypes
Pitakinra/Aeroderm	IL-4	Phase II completed	Aerovance, Berkeley, CA	NCT00676884 Patients >18 years old	Th2/Tc2	All AD phenotypes
Mepolizumab	IL-5	Phase II completed	Glaxo SmithKline, Research Triangle Park, NC	NCT03055195 Patients >18 years old	Th2/Tc2	AD with elevated eosinophils
Tralokinumab	IL-13	Phase II completed	MedImmune, Gaithersburg, MD	NCT02347176 Patients >18 years old	Th2/Tc2	All AD phenotypes
Lebrikizumab	IL-13	Phase II completed Phase III	Hoffman-LaRoche, Basel, Switzerland	NCT02340234 NCT02465606 NCT03443024 Patients >18 years old NCT04250337, NCT04250350 Patients 12–17 years old active	Th2/Tc2	All AD phenotypes
QAW039/Fevipiprant	CRTH2	Phase II completed (drug development programme stopped)	Novartis, Basel, Switzerland	NCT01785602 Patients >18 years old	Th2/Tc2	All AD phenotypes
OC000459	CRTH2	Phase II completed (drug development programme stopped)	Atopix, Carlsbad, CA	NCT02002208 Patients >18 years old	Th2/Tc2	All AD phenotypes
AMG157/tezepelumab	TSLP	Phase I completed	Amgen, Newbury Park, CA	NCT00757042 Patients >18 years old	Th2/Tc2, Th17	All AD phenotypes, prevention of allergic march
MK-8226	TSLPR	Phase I completed	Merck, White-house Station, NJ	NCT01732510 Patients >18 years old	Th2/Tc2, Th17	All AD phenotypes, prevention of allergic march
GBR830	OX40	Phase I completed	Glenmark, Mumbai, India	NCT02683928 Patients >18 years old	Th2/Tc2	All AD phenotypes
KHK4083	OX40	Phase I completed	Kyowa HAkko Kirin, Otemachu, Japan	NCT03096223 Patients >18 years old	Th2/Tc2	All AD phenotypes
QGE031	IgE	Phase II completed	Novartis	NCT01552629 Patients >18 years old	Allergic sensitization	Extrinsic AD, AD in African Americans, AD in Asians, childhood AD
Tofacitinib	JAK1/3	Phase II completed	Innovaderm, Montreal, Quebec, Canada	NCT02001181 Patients >18 years old	Th1, Th2, Th22, IFN-a, IgE class – keratinocyte modulation pruritus differentiation	All AD phenotypes
Baricitinib (LY3009104)	JAK1/2	Phase II completed	Eli Lilly, India-napolis, IN	NCT02576938 Patients >18 years old	Th1, Th2, Th22, IFN-a, IgE class – keratinocyte modulation pruritus differentiation	All AD phenotypes
Upadacitinib (ABT-494)	JAK1	Phase II completed	AbbVie, Lake Bluff, IL	NCT02925117 Patients >18 years old	Th1, Th2, Th22, IFN-a, IgE class – keratinocyte modulation pruritus differentiation	All AD phenotypes
ASN002	JAK/SYK	Phase II completed	Asana BioSciences, Lawrenceville, NJ	NCT03531957 Patients >18 years old	Th1, Th2, Th22, IFN-a, IgE class – keratinocyte modulation pruritus differentiation + Th17	All AD phenotypes
PF-04965842	JAK1	Phase III completed	Pfizer, New York, NY	NCT03349060 Patients >12 years old	Th1, Th2, Th22, IFN-a, IgE class – keratinocyte modulation pruritus differentiation	All AD phenotypes
Crisaborole/Eucrisa Staquis	PDE_4_	Approved by FDA 2016 >2 years FDA 2020 >3 months EMA 2020 >2 years	Pfizer Labs, NY; Pfizer Europe, MA EEIG	NCT02118766 NCT02118792 Phase II completed in children >2 years old	Anti-inflammatory drugs (NSAIDs)	All AD phenotypes
Roflumilast	PDE_4_	Phase II completed	AstraZeneca, Cambridge, UK	NCT01856764 Patients >18 years old	Anti-inflammatory drugs (NSAIDs)	All AD phenotypes
RVT-501	PDE_4_	Active phase I	Dermavant Sciences, Phoenix, AZ	NCT03415282 Patients >2–11 years old	Anti-inflammatory drugs (NSAIDs)	All AD phenotypes
Apremilast/Otezla	PDE_4_	Phase II completed (drug development programme stopped)	Celgene, Summit, NJ	NCT02087943 Patients >18 years old	Anti-inflammatory drugs (NSAIDs)	All AD phenotypes
Ustekinumab/Stelara	IL-12/23p40	Phase II completed	Janssen, Beerse, Belgium	NCT01806662 Patients >18 years old	Th17, Th1, Th22	Intrinsic AD, AD in Asians, childhood AD, EA in patients with chronic AD
CIM331/nemolizumab	IL-31R	Phase II completed	Chugai, Tokyo, Japan	NCT01986933 Patients >18 years old	Pruritus/Th2	All AD phenotypes
BMS-981164	IL-31	Phase I completed	Bristol-Myers Squibb, New York, NY	NCT01614756 Patients >18 years old	Pruritus/Th2	All AD phenotypes
ILV-094/Fezakinumab	IL-22	Phase II completed	Pfizer	NCT01941537 Patients >18 years old	Th22, Th17	Intrinsic AD, AD in Asians, adult EA patients with AD, African American
Secukinumab/Cosentyx	IL-17A	Phase II completed	Novartis	NCT02594098 Patients >18 years old	Th17 (and IL-22)	Intrinsic AD, AD in Asians, children and young adults from AD
MOR106	IL-17C	Active phase II	Galapagos NV, Mechelen, Belgium	NCT03568071 Patients >18 years old	Th17	Intrinsic AD, AD in Asians, children and young adults from AD

*Source*: Czarnowicki et al. (40).

## Summary

Approval of dupilumab for the treatment of patients with AD was a breakthrough in modern approach to the patients suffering from AD. Discovery of IL-4 and IL-13 role (in inflammation process) allowed to create a biological drug, which inhibited the above cytokines leading to significant decrease of AD symptoms while maintaining a good safety profile. Opening the way for research into biological drugs creates hope for the future for more personalized (targeted on phenotype, biomarkers and individual response to treatment) therapy of patients with AD. The next step for Polish patients with AD will be the possibility of gaining access to treatment due to the drug programme on dupilumab.

## Abbreviations:

AD: Atopic dermatitis;
BSA: Body surface area;
CS: Corticosteroids;
CsA: Cyclosporine;
CRTh2: Chemoattractant receptor-homologous molecule expressed on Th2 cells;
DC: Dendritic cells;
EASI: Eczema Area and Severity Index;
ECAP: *Epidemiologia Chorób Alergicznych w Polsce*;
EMA: European Medicines Agency;
FDA: Food and Drug Administration;
FLG: filaggrin;
IGA: Investigator Global Assessment;
ILC: Innate lymphoid cells;
JAK: Janus kinase;
MTX: Methotrexate;
NGF: Nerve Growth Factor;
PDE-4: Phosphodiesterase 4;
SCORAD: Scoring Atopic Dermatitis;
*S. aureus*: *Staphylococcus aureus*;
STAT: Signal transducer and activator of transcription;
SYK: Spleen tyrosine kinase;
TCS: Topical corticosteroids;
TCI: Topical calcineurin inhibitors;
TSLP: Thymic stromal lymphopoietin;
TYK2: Tyrosine kinase 2;
VAS: visual analogue scale.
